# The Effect of Surface Treatments on the Degradation of Biomedical Mg Alloys—A Review Paper

**DOI:** 10.3390/ma11122561

**Published:** 2018-12-16

**Authors:** Marcjanna Maria Gawlik, Björn Wiese, Valérie Desharnais, Thomas Ebel, Regine Willumeit-Römer

**Affiliations:** 1 Helmholtz-Zentrum Geesthacht, Max-Planck-Straße 1, 21502 Geesthacht, Germany; bjoern.wiese@hzg.de (B.W.); valerie.desharnais@sympatico.ca (V.D.); thomas.ebel@hzg.de (T.E.); regine.willumeit@hzg.de (R.W.-R.); 2 School of Computer Science, McGill University, 845 Sherbrooke Street West, Montréal, QC H3A 2T5, Canada

**Keywords:** surface treatments, roughness, Mg-alloys, degradation behavior

## Abstract

This report reviews the effects of chemical, physical, and mechanical surface treatments on the degradation behavior of Mg alloys via their influence on the roughness and surface morphology. Many studies have been focused on technically-used AZ alloys and a few investigations regarding the surface treatment of biodegradable and Al-free Mg alloys, especially under physiological conditions. These treatments tailor the surface roughness, homogenize the morphology, and decrease the degradation rate of the alloys. Conversely, there have also been reports which showed that rough surfaces lead to less pitting and good cell adherence. Besides roughness, there are many other parameters which are much more important than roughness when regarding the degradation behavior of an alloy. These studies, which indicate the relationship between surface treatments, roughness and degradation, require further elaboration, particularly for biomedical Mg alloy applications.

## 1. Introduction

The study of Mg as degradable biomaterial for implants is an advanced research area. A second operation to remove the implant after bone healing can be avoided [1,2,3,4,5,6]. Mg is naturally available as trace element in the body, and is thus non-toxic and biocompatible [7,8,9]. Implant processing is feasible due to the ductility and workability of Mg [10]. Strength and toughness are higher than of polymer implants, which is beneficial for load-bearing implants [11,12]. Mg alloys are reported to show improved osseointegration and bone implant strength compared to permanent Ti alloys [13,14]. In particular, Mg alloys are suitable as biodegradable implant materials [1,15,16,17,18,19]. Mg is able to degrade in aqueous solutions with the formation of magnesium hydroxide and hydrogen [20,21,22,23,24]. In particular, aqueous salt solutions containing ions including chlorides or sulphates, with the exception of alkali metals or alkaline metal containing solutions, are able to dissolve the protective magnesium hydroxide layer, leading to enhanced degradation [24,25,26,27,28]. In order to improve the mechanical properties of Mg, elements are added to tailor, for example, its tensile strength and ductility. Thus, it is possible to produce implants that have tailored mechanical properties to use it as temporary bone fixation. However, when alloying and processing the material, impurities like Fe, Ni, and Cu or phases with a high electrochemical potential difference are found at or near to the surface of the material, which increases the degradation rate through galvanic corrosion [23,29]. For the application of biodegradable Mg implants to become feasible in the future, two different objectives must be met in order to achieve usable degradation behavior.

One objective is limiting the degradation rate of the initial state of the alloy, which, as explained later, is related to the amount of hydrogen evolution. The deeper and rougher the surface morphology, the more hydrogen gas will be produced [9]. An overly fast degradation with gas evolution in the initial state leads to degradation of the mechanical integrity. Excessive gas evolution can also modify the bone remodeling process and impair the consolidation of bones [30]. However, relatively strong hydrogen evolution is crucial for cell adherence and implant-bone integration [21,31,32]. Aqueous salt solutions including chloride ions, like those found in the human body fluids [33], increasing decomposing of Mg(OH)_2_, release OH^−^ and raise the pH [34]. Besides hydrogen production, a local alkalization might provoke necrosis [9].

The second objective is to control the degradation rate of implants during the healing time. The required degradation rate depends on the application with lifetime and stability of the implant and the potential of the surrounding tissue to tolerate pH changes and high ion concentrations. It is reported that the properties of the material, e.g., crystallographic orientation [35,36,37], microstructure [21,38,39,40,41,42,43,44,45,46,47,48], grain size [41,49,50,51,52,53], secondary phases [51,54,55], contamination [38,40,56], and deformation [38,41,57,58,59,60], affect the degradation behavior, as well the environment, e.g., the immersion medium [61,62,63,64]. It is possible to control the degradation behavior of Mg alloys using chemical, physical, and mechanical surface treatments [27,32,65,66,67,68,69]. Additionally, surface uniformity has been shown to decelerate degradation [70]. Surface morphology can differ despite identical roughness parameters, and also affects the degradation process [39]. Studies have shown that surface roughness can affect the initial degradation [71], the degradation rate [38,71,72,73,74,75,76,77], degradation resistance [73,78,79,80,81,82], pitting behavior [38,71,72,83], bone integration [84,85,86], cell adherence [21,74,87,88], cell proliferation [88,89,90,91], and cell differentiation [92]. Besides roughness, surface unevenness can also influence the adhesion of cells [73]. In some cases, a smoother surface will reduce the degradation rate [72,74,93]. However, this behavior has been contradicted in other studies [73,79,81,82,94,95].

The aim of this review is to show the correlation between surface treatment, roughness, and the degradation behavior of Mg alloys in order to define meaningful roughness values and suitable surface treatments for biodegradable Mg implants. An overview of studies mentioning surface treatments, roughness, and degradation is given in Table 1, Table 2, Table 3, Table 4 and Table 5.

## 2. Mechanical Surface Treatments 

### 2.1. Grinding and Polishing

**Figure 1:**
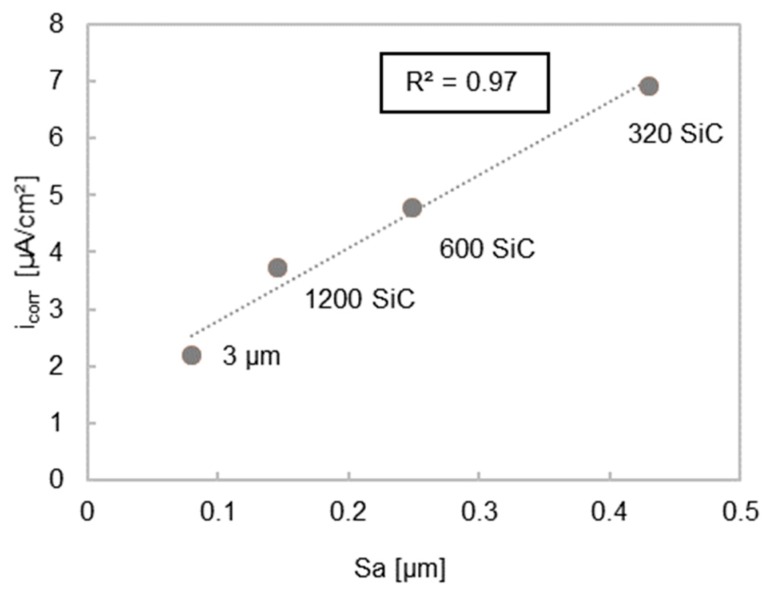
Graph shows a linear relationship between Sa and i_corr_ after grinding and polishing. R² is the coefficient of determination, which assess the linear mutual dependence of x and y. R² = 1 defines the highest linearity [97]. Sa and i_corr_ values were obtained from [96].

The degradation behavior of sand-cast, ground, and polished AZ91 alloys were investigated by Walter and Kannan [96]. The use of a grinding paper with increased grit size decreased the surface roughness (Table 1, Ref. [96]). Three methods were used to evaluate the relationship between pitting and roughness: a 24 h immersion test in a 0.5 wt.% NaCl solution, 1 h of potentiodynamic polarization (PDP) and 1 h of Electrochemical Impedance Spectroscopy (EIS). For the polished samples, no inductive loop after EIS was observed. A low inductive loop is related to a low or negligible amount of surface pitting. This was found for all ground samples. Thus, it is suspected that no pitting will occur on polished surfaces due to a higher passivation. As a consequence, passivation is reduced for higher surface roughness values. The polarization curves in Ref. [96] show that a higher anodic current, as indicated by the current density i_corr_, is produced with greater surface roughness (Table 1, Ref. [96] and Figure 1).

In particular, the polarization curve of material ground with 320 grit paper in Ref. [96] exhibits a strong increase in anodic current, which suggests a high number of pits being formed. The surface appearance was analyzed by scanning electron microscope (SEM) after immersion for 24 h and after galvanostatic testing. Numerous pits were observed after immersion when the 320 grit size paper was used, which confirms the results from the electrochemical testing. Less pitting was seen to occur when using a finer grinding paper. In the case of paper with a 1200 grit size, no localized pitting was found after testing. Surprisingly, more pits were observed on polished samples after galvanostatic testing. This is likely to have been caused by a high anodic current which reduces passivation for all surface treatments. Walter and Kannan [96] concluded from these experiments that roughness does affect the passivation layer, but does not directly affect the likelihood of pitting. After removing the passivation layer, pitting occurred for all surface roughness values as seen by SEM [96].

Walter et al. [71] also investigated the correlation between the degradation and surface roughness Sa (the arithmetic mean height within a sample area, three dimensionally determined roughness) [98] for samples which were ground using 120 SiC grit size paper (Sa = 973 nm) and samples that had been ground using 2500 grit size paper, followed by polishing with a 3 µm diamond paste (Sa = 22 nm). The samples were cleaned with acetone and ethanol. The degradation behavior was characterized under simulated body fluid (SBF) using EIS. The results for both surface finishes exhibited similar tendencies. The ground and the polished samples showed a mid-frequency capacitive loop at the beginning of testing [71]. A mid-frequency capacitive loop corresponds to a passivation layer [96]. For the ground sample, a mid-frequency capacitive loop was observed for the first 2 h and was then followed by an inductive loop at low frequencies. The polished samples had inductive loops at low frequencies after 4 h, which confirms that passivation layers on smoother surfaces last longer [71]. In general, an inductive loop implies surface pitting [99]. The polarization resistance was present for a maximum of 3 h for the polished sample in contrast to a maximum of 2 h for rougher ground surface. This observation agrees with the assumption that polished samples have a higher passivation. Thus polishing samples reduces the degradation behavior, especially at the beginning of immersion. The SEM results support these findings. After 2 h immersion, a general degradation for both finishes was observed. After 6 h, the rougher surface clearly showed more pitting, while the few pits seen on the polished surface indicated the start of pit formation. Pitting was studied for both surfaces after 12 h immersion. The initial pitting of the ground surface had progressed further compared to the polished finish. Walter et al. [71] explained this observation as a local pH drop caused by deep valleys in the rough surface. Additionally, the passivation layer of the rough surface broke up earlier than the smooth surface [71].

Alvarez et al. [94] found that polished AE44 samples encouraged more pitting compared to semi-polished samples in an immersion test. At the beginning of degradation, polished samples exhibited a higher pit volume compared to semi-polished samples. However, semi-polished samples had higher pit radii. The smoother surface of the polished sample and its related chlorine absorption capacity is given as a possible explanation. This behavior is distinct from reports that report that rougher surfaces on steel [75,76] and aluminum [77] lead to faster degradation and more pitting. Walter and Kannan [96] suggested a change of the passivation layer provoked by shifting the local pH, initiated by aeration of the solution, as a reason for this behavior [96]. Pitting for both conditions leads to intergranular degradation after several hours. However, the start of intergranular degradation of the polished samples started earlier than for semi-polished samples [94].

Lorenz et al. [74] showed that surface roughness does not only influence the degradation resistance of pure magnesium; it also affects the cell (HeLa cells/GSP-C12 mouse fibroblasts) adhesion on the surface of Mg. For this study, discs were prepared with 600 paper grit size, a combination of 6 µm diamond paste, and an ethanol/glycerol solution. Sample cleaning was performed using an ultrasonic bath filled with ethanol for 3 min. In order to analyze the effects of surface morphology on cell adherence, one series of samples was immersed in 1 mol NaOH for 24 h and another series in modified simulated body fluid (M-SBF) at 37 °C for 5 d. Afterwards, the samples were flushed with ethanol and dried in air. The roughness increased after immersion in both solutions, but especially for the modified simulated body fluid (M-SBF) solution. pH measurements were also carried out on Mg samples degrading in a Minimum Essential Media (MEM) that included fetal bovine serum (FBS). A pH of 8.96 was observed for the M-SBF treated Mg samples and was higher compared to the other treatments after 2 h (Table 1 and Table 4, Ref. [74]). The thicker Ca/Mg phosphate layer after M-SBF immersion does not protect the Mg sample due to its porosity, but the corrosion resistance increased by a factor of five compared to the untreated samples. In contrast, the cell density is higher compared to the polished and NaOH treated samples. The increase of the roughness by immersing in M-SBF improved the cell adhesion. The medium alkalization of the M-SBF samples is only suitable for short term applications. The smooth surface of the polished samples exhibits nearly no cell adherence and degrades very quickly. The passivation of Mg with NaOH reduces degradation, but cell adhesion is lower compared to M-SBF immersion [74].

Liu [61] compared the cell adherence of rolled pure Mg foils with an oxide layer and on ground foils without an oxide layer. He also studied the effects of roughness and degradation in Dulbecco’s Modified Eagle’s Medium (DMEM) and deionized (DI) water. The smoother ground samples varied by only 1.2% (oxide layer 13.6% and on ground foils 14.8%) in cell density from the rough oxidized samples (Table 1 and Table 2, Ref. [61]). As such, it can be assumed, that surface roughness did not affect the cell density. No correlation between roughness and degradation rate was found, though ground samples in DMEM showed a slower degradation rate compared to oxidized samples. The opposite behavior was observed in DI water [61].

In contrast to the studies carried out by Liu [61] and Lorenz et al. [74], it was reported by Johnson et al. [21] that ground Mg-4Y samples demonstrated a better cell attachment than samples with an electrical discharged machined (EDM) surface. The roughness of the surfaces could be a possible explanation, as it was found that rough surfaces degrade faster than smooth surfaces [21,72,93]. Mg-4Y exhibits a contrary degradation behavior as pure Mg [61]. The ground surface leads to a lower mass loss in DI water which is opposite to the higher mass loss in DMEM (Table 1 and Table 2, Ref. [21]). This effect is not thought to be due to roughness, but rather, from a different evolution of the pH under the different testing conditions.

Song and Xu [38] investigated the effect of tempering (HT), sandblasting, grinding, and etching on the degradation resistance of the alloy AZ31. Tempering and sandblasting reduced degradation resistance, while grinding or acid etching as a cleaning procedure decreased weight loss and hydrogen evolution. Heat treatment led to the precipitations of large Al-Mn-Fe particles which deteriorated the degradation resistance [38]. In addition to impurities, it is also known that roughness influences the degradation rate [100]. The roughness Ra (two-dimensionally determined roughness, arithmetic mean deviation of the roughness profile) and the hydrogen evolution of ground samples were very low compared to sandblasted samples (Table 1 and Table 2, Ref. [38]). Sandblasting led to a very rough surface, accompanied by micro stresses in the surface layer. The Fe impurities rather than this surface roughening increased the degradation rate. Grinding the surface removes contaminations and leads to a slower degradation rate. Though ground surfaces are smoother than etched, the effect on the degradation is not as significant as removing a significant amount of Fe [38].

Zhao and Zhu [6] investigated, in addition to collagen monomer concentration, pH, and assembly time, the influence of ground surfaces on collagen fibril formation. They tested different surface finishes and the collagen formation with subsequent cell attachment. They ground Mg and AZ31 discs with 180 (Ra = 1.89 µm), 800 (Ra = 0.29 µm) and 1200 (Ra = 0.15 µm) SiC paper and apply 50 µL of 200 µg/mL D-phosphate-buffered solution (DPBS) diluted collagen solution for 2 h on the samples. By SEM they observed the morphology of collagen fibers for every surface finish and alloy. A clear difference of collagen formation was visible from roughest to smoothest surface for both alloys. While the collagen formation on both alloys for both smoothest surfaces was comparable, the roughest surface of AZ31 showed less dense structure in contrast to Mg. The roughest surface of both alloys adsorbed the highest amount of collagen after 2 h, while the smoothest surface showed the lowest adsorbed amount. This trend was more distinct for Mg compared to AZ31. Cell attachment observations after 2 h lead to the assumption that cells were more attached on collagen treated and smoother samples in contrast to the roughest surface finish. The roughest surface finish with a more fiber woven structure and highest collagen adsorption also showed in another Fluorescent live/dead cell analysis that, after one day, the collagen structure on a rough surface is more detrimental to cell density, independent of the alloy [6]. Nudelman et al. [101], reported a correlation between collagen and cell attachment [101]. In contrast to Nudelman et al. [101], Zhao and Zhu [6] evidenced a decrease in cell density with higher collagen adsorption. For this reason, it is assumed, that more collagen does not always result in a stronger cell attachment. In reference to roughness, this study shows an effect on the collagen formation which influences the cell density indirectly [6].

### 2.2. Burnishing

A comparison between ground and burnished sample degradation was performed using hydrogen evolution, PDP, and white light interferometry on the AZ31B alloy [39]. Ground and burnished (dry and cryogenic) samples had a very similar roughness before degradation. Burnishing was performed using a severe plasticity burnishing (SPB) process. Cryogenic burnishing is distinguished from dry burnishing by the use of liquid nitrogen. After degradation, the morphology of the ground samples differed from the burnished samples. Thus, roughness will not be the only factor to influence the degradation behavior. In addition, grain size and basal texture had an influence on the uniformity and amount of degradation. Dry and cryogenic burnishing decreased hydrogen evolution over a 7 h period with respect to ground surfaces. Both burnishing processes lead to a smoother finish with reduced pit depth and pit volume compared to the 4000 grit size paper treatment immersion test. The PDP analysis shows the same trend for both burnished surfaces with a higher degradation resistance, indicated by a broader capacitive loop [39]. The crystallographic orientation and grain refinement has to be considered, as well as the surface roughness [38]. In this report, the surface roughness did not affect the degradation, in disagreement with the prediction of Song and Xu [38]. Moreover, a small grain size and a strong basal texture led to a higher degradation resistance [39].

### 2.3. Machining

Turned, threaded and sandblasted Mg-0.8Ca samples were examined and tested in vivo [93]. Smooth (Ra = 3.65 µm) turned and threaded samples exhibited the best interlocking connection between the bone and implant. Rough (Ra = 32.7 µm) sandblasted rods degraded most rapidly with the highest number of visible gas bubbles. Turned surfaces led to the lowest gas evolution and decomposition in these studies [87,93]. Despite a similar integration of threaded and turned implants into the bone tissue, threaded implants showed a non-uniform bone resorption at the thread edges [93]. This is in agreement with the findings of Walter et al. [71], which may be explained by local variations in pH.

**Figure 2:**
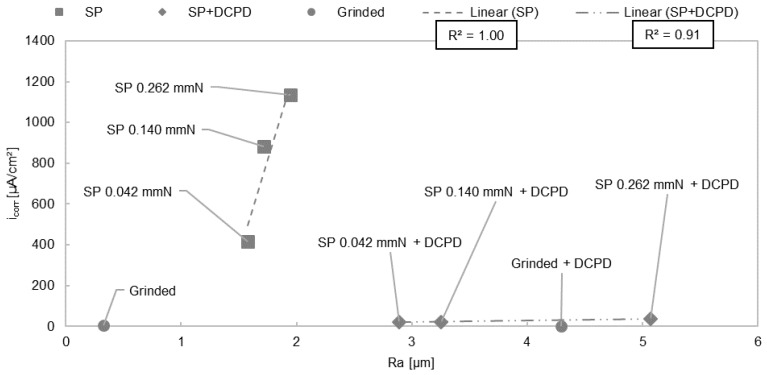
Diagram current density i_corr_ against roughness Ra for ground, shot peened, and shot peened + DCPD coated samples. A non-linear R² relationship between Ra and i_corr_ is shown by comparing all conditions (ground/SP/SP+DCPD) together. Linear R² is plotted for only shot peened or only shot peened and coated samples. A trend of linearity can be seen only for roughness values arising from same surface treatments. Ra and i_corr_ values were obtained from [102].

Mhaede et al. [102] reported a relationship between roughness and corrosion resistance. For the degradation test in 0.9 wt.% NaCl solution, eight different conditions of AZ31 alloy were prepared. Samples were either ground or shot-peened with 3 different Almen intensities (saturation value of residual arc height of an Almen strip, established by John Almen) [102,106,107], ground and coated, or shot-peened with 3 different Almen intensities and coated without prior grinding. Shot peening was performed with ceramic shot which had a diameter of 850 µm (Z850). The dicalcium phosphate dihydrate (DCPD) coating used was produced by electro-deposition of samples in a 0.1 mol Ca(NO_3_)_2_ 4H_2_O + 0.06 mol NH_4_HPO_4_ solution. In Table 2, Ref. [102] it is shown that the current density i_corr_ for the shot-peened (SP) samples was increased compared to the other conditions in Table 4, Ref. [102]. It has been shown that having a rough surface after shot-peening affects i_corr_ (Table 2, Ref. [102] and Figure 2), as the resulting greater surface area increases the surface reactivity [102]. However, it is not possible to relay Ra linear to i_corr_ (Table 1, Table 2 and Table 4, Ref. [102] and Figure 2) for all surface finishes due to the protective properties of the DCPD coating compared to only shot peened samples. The linear relationship between Ra and i_corr_ for the shot-peened and shot-peened/coated samples (Figure 2) agrees with the study of Walter and Kannan [96], whereas linear correlation was observed for only the ground samples. However, it should be noted that higher deformation and internal stress, arising from higher Almen intensities, could also affect the degradation behavior. 

Denkena and Lucas [108] studied the surface and subsurface properties after turning and deep rolling a Mg-3Ca alloy. Three different conditions for each machining process were investigated. With regard to turning, the roughness Rz (distance from deepest valley to highest peak within sample length from a linear measurement) [109] decreased from around Rz ~ 4.48 µm to Rz ~ 3.75 µm after increasing the cutting speed from 10 m/min to 100 m/min at constant cutting depth and feed rate. By reducing the feed rate from 0.1 mm to 0.05 mm at a constant cutting speed (100 m/min) and cutting depth (0.5 mm), the roughness (Rz ~ 2.17 µm) was reduced (Table 2, Ref. [108]). After deep rolling with different rolling forces (F = 50 N, 200 N, 500 N) and constant rolling speed (25 m/min) and feed rate (0.1 mm), no significant change in roughness (Rz ~ 0.91–1.26 µm) occurred (Table 2, Ref. [108]). Degradation tests were performed in 0.9 wt.% NaCl solution and the hydrogen gas evolution was measured. The mass loss was calculated from the amount of hydrogen produced and a correction factor. The degradation rates for turning with higher roughness were greater compared to the degradation rates after rolling. However, for turning, the condition with the highest roughness showed the lowest mass loss compared to the smoother samples. For deep rolling, the condition with the lowest rolling force led to the highest, while not signifying mass loss (calculated from hydrogen generation) after around 240 h exposure time compared to conditions with higher rolling forces and comparable Rz values. No significant correlation between roughness and mass loss was found. High residual compressive stress was reported to reduce the degradation rate by about 100 times [108], and the degradation results were comparable with the results from high speed dry milled Mg-0.8Ca with the lowest roughness [110]. 

The influence of machining and deep rolling on Mg-3Ca and Mg-0.8Ca was analyzed by Denkena et al. [111], and the results were compared to those of Denkena and Lucas [108]. Only 3 conditions per alloy were tested. The turning was carried out with a cutting speed of 100 m/min, cutting depth of 200 µm, and a feed rate of 0.1 mm. Two deep rolling conditions were studied with rolling forces of 50 N and 200 N and the same cutting speed and feed as described by Denkena and Lucas [108]. The roughness Rz after turning and deep rolling was comparable for each alloy for every machining process. The roughness Rz after turning was about 4 µm, while Rz for deep rolling resulted in a lower Rz of between 0.44–0.76 µm (Table 2, Ref. [111],[108]) compared to Denkena and Lucas [108]. The corrosion behavior was tested by hydrogen evolution and performed in a 0.9 wt.% NaCl solution and µ-CT. It was shown that turned Mg-3Ca with the highest Rz resulted in the highest hydrogen evolution (~ 20.2 mL/cm² after 29 h) and greatest degradation in µ-CT compared to deep rolled samples (~0.76–1.27 mL/cm² after 29 h). For the Mg-0.8Ca alloy the hydrogen evolution (~5.42–6.22 mL/cm² after 29 h) showed no significant dependence on the method of machining. From these investigations, it is possible to say that roughness had no influence on the degradation behavior. Rather than roughness, a high compressive stress and the Mg_2_Ca phase in the Mg-3Ca alloy was reported to affect the degradation behavior [111].

## 3. Chemical Surface Treatments and Coatings

### 3.1. Acid Etching

The treatments reported by Supplit et al. [95] indicated that it was possible to improve the degradation resistance of rolled AZ31 alloy by pickling with different acids like acetic acid, phosphoric acid, nitric acid, and hydrofluoric acid [95]. Acid pickling, especially with acetic acid decreased the degradation rate from 7.17 mg cm^−2^ d^−1^ (rolled condition) to 0.70 mg cm^−2^ d^−1^. The second best etching method was found to be phosphoric acid. The degradation rates were determined by measuring hydrogen gas evolution in 5 % NaCl. A rougher surface after pickling with acetic acid was observed when compared to the other etching solutions. The samples with the lowest degradation rates had rougher surfaces, an observation that contradicts the findings of Nguyen et al. [72].

Organic acids like acetic, citric, or oxalic, and inorganic acids like phosphoric, nitric, and sulfuric acid were used to treat AZ31 alloy by Nwaogu et al. [40]. The aim was to remove contamination and impurities from resulting from rolling. After etching, 1–20 µm was removed from the surface. It was observed that more material was removed as the etching time increased. A roughness analysis showed that the roughness value Ra after etching is higher than Ra of rolled samples. Removing 5 µm of material generally reduced the number of Ni impurities. However, Fe impurities still remained at the surface even after material had been removed. To determine the degradation behavior, a 48 h salt spray test was used as a screening test. The lowest degradation rates were obtained from samples with the lowest impurity levels which had the greatest amount of material removed. Acetic acid-etched samples had the slowest degradation rates. EIS measurements supported the finding that acetic acid etching leads to the resistance, due to having the highest polarization resistance (Rp) [40]. This finding is in agreement with the results obtained by Supplit et al. [95] and Nwaogu et al. [40], who showed that a low impurity level and a 5 µm etching depth improved the degradation behavior. When more than 5 µm of material was removed, the surface became rougher [40].

The change in roughness after inorganic acid etching confirmed this finding [56]. For sulfuric acid etching, Ra (>2 µm) was much higher compared to other inorganic etching solutions when 7 µm of material had been removed. In addition, sulfuric acid etching leads to a lower degradation resistance in spite of the resulting low level of impurities. Degradation of sulfuric acid-etched material mostly results from galvanic degradation initiated by second phases. Though the effect of roughness were not the main focus in these investigations, nitric acid etching showed a high degradation resistance for a surface with an initially uniform roughness distribution and low roughness value [56]. Thus, the roughness of a sample after etching could also be a parameter which has to be considered in order to determine the full degradation behavior.

Gawlik et al. [57] measured the roughness after acetic acid etching with various combinations of acid concentration and immersion time. The surface roughness Sa increased after etching compared to the milled surface and varies with different conditions. After 30 days immersion, the same degradation rate was determined for all etched conditions (Table 3, Ref. [57]), in spite of different Sa and Sq (root mean square value of surface deviations) [98] values after etching. This leads to the conclusion that the initial roughness of the sample has no long-term effect on degradation. The varying surface morphology and near-surface deformation arising from milling also affected the degradation rate [57].

Similarly to Nwaogo et al. [40,56], Song and Xu [38] described that Fe impurities accelerate the degradation of the AZ31 alloy. As such, as-received samples and heat-treated samples have lower degradation resistance compared to ground and sulfuric acid etched samples, due to Fe particles remaining on the surface. Sulfuric acid etching roughens the surface much more than grinding, but both conditions lead to a similar degradation rate. Thus, the roughness of the etched samples itself does not contribute to the degradation rate. Acid cleaning removes contamination and the deformation zone arising from processing, and thus directly impacts the degradation behavior [38].

Gray-Munro et al. [70] also tested acetic treatments on AZ31 alloy. Gray and Luan’s study [68] described that a strong passive oxide layer was formed during the etching process when compared to the as-received state. Gray-Munro et al. [70] found that as-received samples have a greater non-uniform morphology in comparison with phosphoric acid etched samples. Phosphoric acid treated samples showed a lower degradation rate of 8.27 mg/d compared to non-etched samples (~31 mg/d). Additionally, the modified surface after etching improves adhesion and minimizes the porosity of coatings [70]. 

### 3.2. Coatings

Gray-Munro et al. [70] discovered that biomimetic calcium phosphate coatings (Ca/P) and polymer coatings after phosphoric etching led to a uniform morphology, which in turn led to a uniform degradation over the surface of the AZ31 alloy. The degradation rates of 6.17 mg/d (Table 4, Ref. [70]) for a polymer poly(L-lactic acid) (PLA) coating and of 3.83 mg/d for a poly (desaminotyrosyl tyrosine hexyl) (DTH) carbonate coating are compared to etched samples with rates of 8.27 mg/d (Table 3, Ref. [70]) (sample size: 1 mm thick foil, 10 mm × 20 mm). The degradation rate (7.27 mg/d) resulting from the Ca/P coated samples did not strongly differ from the only etched alloys [70]. However, the Ca/P-coated Mg alloy exhibited non-toxic and biocompatible properties. Ca/P enhanced the osseointegration and bioresorption of the alloy in a physical environment [112,113,114,115,116], which is why a Ca/P coating is more favorable compared to phosphoric etching.

Bakhsheshi-Rad et al. [104] performed potentiodynamic polarization (PDP) tests and immersion tests in SBF (Kokubo solution) on polished Mg-0.5Ca-6Zn samples with and without a coating. The coatings tested were a fluoride conversion coating, a dicalcium phosphate dihydrate/magnesium fluoride (DCPD/MgF_2_) coating, and a nano-hydroxyapatite/magnesium fluoride (nano-HA/MgF2) coating. A higher root mean square roughness (Rq) was measured for polished Mg-0.5Ca-6Zn samples with either a DCPD/MgF2 coating (Rq = 395 nm) or a nano-HA/MgF2 coating (Rq = 468 nm). The Rq for polished Mg-0.5Ca-6Zn (Rq = 210 nm) samples without coating and polished Mg-0.5Ca-6Zn samples with a fluoride coating (Rq = 280 nm) were somewhat lower. As seen in Table 4, Ref. [104], hydrogen evolution, i_corr_ and the degradation rate declined as the surface roughness increased, in contrast to studies of Walter and Kannan [96] and Mhaede et al. [102]. As shown in Table 4, Ref. [104], the degradation rate after coating compared to non-coated Mg alloy in Table 1, Ref. [104] is about a factor of 60 smaller, even though Rq only differs by 70 nm [104]. Thus, the protective coatings have a greater influence on corrosion than the Rq values. 

Pompa et al. [103] investigated the morphology, surface roughness, cell viability, and degradation rate of ground and anodized AZ31B, AZ91E, and ZK60A alloys. Grinding was performed with a 1200 grit size grinding paper. Anodization was carried out using a mixture of alcohol and organic acid. The surface roughness increased dramatically from Sa = 29.76 nm to Sa = 204.81 nm after anodization for the AZ91E alloy. The anodization of AZ31B (Sa = 48.58 µm) and ZK60A (Sa = 78.30 µm) did not change the roughness significantly. It was shown that anodizing decreased the degradation rate compared to a ground surface. No correlation between surface roughness and degradation rate was found. This may be due to corrosion resistances being similar for all anodized surfaces despite their variation in roughness [103].

In the study of Chiu et al. [80], AZ31 plates were arc sprayed and hot pressed. Anodizing with oxalic acid was then performed [80]. The current density i_corr_ decreased after a combination of spraying and hot pressing or additional anodizing (Table 4, Ref. [80]) as compared to uncoated sandblasted samples (Table 2, Ref. [80]). Hot pressing decreased the surface roughness, which seems to improve the acid treatment afterwards. No correlation between roughness and degradation resistance was reported [80].

Yoo et al. [81] studied the effect of roughness on the degradation resistance of a plasma electrolytic oxidation (PEO) layer on a AZ91 alloy. Surfaces with various Ra were prepared by grinding and polishing. Afterwards, all samples were coated using the same PEO process. Due to the differing roughness values of the ground and polished surfaces, the coating differentiated in pore size as well. This affects the degradation process. The current density increased with higher initial Ra (Table 4, Ref. [81]). A salt spray test also showed that the amount of pitting increased with higher Ra after 120 h, which indicates that the surface roughness before PEO indirectly influences the degradation resistance [81].

Cho et al. [73] compared the degradation resistance of PEO coatings on AZ91 alloy for different amounts of potassium pyrophosphate in the electrolyte. The size of the pores increased as the amount of potassium pyrophosphate was increased. It was also reported that the surface roughness increased with increasing pore size. There was a trend between pore size, surface roughness, and i_corr_ for additions of potassium pyrophosphate (Table 4, Ref. [73]). The PEO coating for the potassium pyrophosphate-free electrolyte exhibited the lowest degradation resistance compared to the rougher coatings [73].

In contrast to Cho et al. [73], Hwang et al. [79] compared PEO coatings on AZ91 alloy with and without potassium fluoride in the electrolyte. In addition to varying the surface roughness, the evolution of the degradation resistance with coating time was also examined. The surface roughness increased for longer coating times. The roughness of the coated samples, after dipping in the potassium fluoride containing electrolyte, was higher compared to coatings dipped into potassium fluoride free electrolyte. The roughness increases due to pore size enlargement as reported by Cho et al. [73]. The degradation resistance of the coatings when exposed to potassium fluoride-containing electrolyte was higher than for potassium fluoride-free electrolyte. Hwang et al. [79] explained that the oxide thickness is the reason for the improved degradation resistance, and did not assess the influence of roughness on the degradation resistance, as investigated in Hwang et al. [82]. The effect of the PEO coating roughness on the degradation behavior was examined in Hwang et al. [82] with PDP and three different coating surface roughness Ra values. The surface roughness increased with increased pore size, as was also seen in Hwang et al. [79] and by Cho et al. [73].

### 3.3. Ion Implantation

Jamesh et al. [105] implanted Si ions from a plasma on polished WE43 plates. Atomic force microscope (AFM) measurements after polishing and Si implantation showed that the surface became smoother after the ion implantation process. The smoother Si-implanted surfaces had improved degradation resistance. However, the roughness did not vary enough between the polished and Si-implanted surface types to obtain a correlation between roughness and degradation [105].

Zhao et al. [88] reported a slower degradation rate for Mg-Ca and Mg-Sr alloys after ion implantation (Zr and O ions) onto their surfaces. After the surfaces were implanted, measurements determined that the surfaces were uniformly rough. The roughness increased after implantation for both alloys (compare Table 1 and Table 5, Ref. [88]). The current density i_corr_ decreased for surfaces with higher roughness, and the cell adherence and proliferation improved [88].

## 4. Summary of the Influence of Roughness on Degradation

### 4.1. Mechanical Surface Treatments 

Using grinding papers with higher grit size and/or polishing reduced surface roughness and reduced pitting during the degradation tests [71,96]. These results differ from those in studies by Alvarez et al. [94], where polished samples encouraged pitting compared to semi polished surfaces [94]. Some papers reported that roughness affects cell adherence to the surface [21,74]. However, one study showed that cell adherence was not influenced by surface roughness [61]. Some studies showed that roughness did not affect degradation [39,102,108,111]. In contrast, it was demonstrated that a linear relationship existed between roughness and degradation if the surface treatments were comparable (Figure 1 and Figure 2) [72,96,102].

### 4.2. Chemical Surface Treatments and Coatings

Some studies investigated the correlation between etched AZ31 alloy samples and degradation behavior [38,40,56,70,80,95,103]. In studies by Chiu et al. [80], Supplit et al. [95], Nwaogu et al. [40,56], and Gray Munro et al. [70], it was found that acetic, nitric, and phosphoric acid surface treatment improved the degradation resistance. In a report by Song and Xu [38], sulfuric acid was shown to enhance degradation, contrasting the study by Nwaogu et al. [56]. The roughness after etching was not reported to affect degradation. Etching had positive effects on the surface as it removed contamination and manufacturing marks, resulting in a homogenous morphology [38,40,56,70]. Ca/P- and polymer coatings also led to a more uniform morphology which improved the overall degradation resistance compared to as-received samples [70]. Further investigations of the correlation between coatings and degradation behavior were performed by Yoo et al. [81], Cho et al. [73], and Hwang et al. [79,82]. They studied the influence of PEO on AZ91. They found that a PEO coating increased the surface roughness. In all reports except for that of Yoo et al. [81], the rougher surface resulted in a greater degradation resistance. Guo and An [78] reported, as did as Yoo et al. [81], Cho et al. [73], Hwang et al. [79,82], and Duan et al. [117], that coatings affect the surface roughness. Additionally, ion implantation is one technique that can be used to smooth the surface and increase the degradation resistance [105]. However, Zhao et al. [88] determined that the degradation rate slowed as the roughness increased after ion implantation.

## 5. Discussion

### 5.1. Suitable Roughness Values for Biodegradable Mg Implants

**Figure 3:**
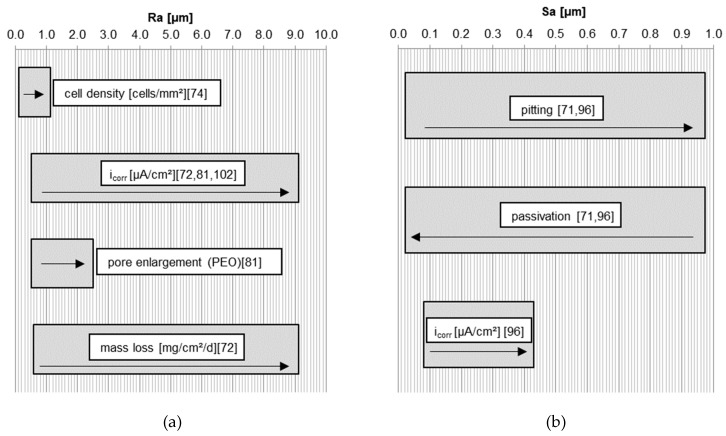
Trend of properties: (**a**) in relation to roughness Ra. The arrow shows the change of properties depending on the roughness according to [72,74,81,102]; (**b**) in relation to roughness Sa. The arrow shows the change of properties depending on the roughness according to [71,96].

Nguyen et al. [72] investigated the influence of roughness on i_corr_ after 6 hours degradation in HBSS (Hank’s Balanced Salt Solution) of pure Mg with indirect solid free form fabrication (SFF). After SFF, no postprocessing is necessary, which enables the production of different degrees of surface roughness with the same surface properties. They avoided the influence of different alloy compositions and surface treatments. It was shown that an increase in Ra led to an increase in i_corr_ and mass loss (Table 2, Ref. [72] and Figure 3a). Some reports concerning surface treatments established that there was no direct influence from the roughness on the degradation behavior. The roughness values from these reports are described using two-dimensional values such as Ra, Rq, and Rz, or three-dimensional parameters like Sa, which cannot be compared directly. The difference between macro-roughness and micro-roughness is also not defined. Macro roughness describes the height distribution which comes from a production process like sawing. As such, the macro-roughness of the sample is not going to influence the degradation process in the same way as micro-roughness. Macro-roughness is accompanied by the subsurface stress that results from production and machining, and which has also been reported to affect degradation [38]. Rougher surfaces influenced the pore enlargement of PEO coatings (Figure 3a) which indirectly controls the degradation rate [81]. 

There was also a trend of increasing current density when using different grinding papers as seen by Walter and Kannan [96] (Figure 1 and Figure 3b) and using different Almen intensities during shot peening (Figure 2, Figure 1 and Figure 3a) [96,102]. In general, higher roughness diminishes the passivation layer and raises the probability of pitting (Figure 3b). Initial pitting effects are only noticeable within the first six hours [71]. As such, it is suggested that roughness has no long-term effects on the degradation as the morphology changes during immersion in aqueous solutions. However, roughness can influence the initial degradation due to greater peak-to-valley height differences, which results in a higher anodizing surface area [102] with a lower pH solution inside the valleys [71]. This roughness effect fades after a short time as the higher surface peaks are eroded away. A more rapid degradation accelerates this process. Even if roughness has a noticeable effect at the start of degradation, as the surface flattens with time it will quickly become insignificant, as seen in the long-term experiments of Gawlik et al. [57]. A correlation of higher cell adherence with higher roughness for pure Mg was reported in [74] (Figure 3a). Cell toxicity and cell adherence have been mainly tested for Sa and Ra values around 1 µm and below. Some reports about osseointegration showed that the connection between dental implants and bone improves when using implants with rougher surfaces introduced by surface modifications [84,118]. One study reported that a roughness between 1–2 µm led to the best connection between a permanent Ti dental implant and bone [119]. Höh et al. [93] could not confirm a trend relating higher roughness to greater bone implant connectivity for biodegradable Mg-0.8Ca alloys. However, in vivo experiments in rabbits showed that sandblasted cylinders with a higher magnitude of roughness (Ra = 32.7 µm) led to strong gas evolution and material decomposition [93]. Mechanical integrity cannot be obtained if the initial degradation is too rapid. Significant hydrogen evolution hinders cell adherence and thus the formation of a good bond between the bone and implant. The required roughness for cell adherence depends on the kind of cells and the necessity of cell adherence. Stronger cell adherence resulting from higher roughness is needed for osseointegration for example, whereas smoother surfaces are preferred in stent applications where cell adhesion is less important. The influence of roughness on the degradation and cell adherence in in vitro and in vivo experiments is not possible either, due to different experimental set-ups and durations. The difficulty in comparing data of in vitro and in vivo testing is reviewed by Sanchez et al. [120]. From all of these studies, depending on the application, roughness values above Sa or Ra = 0.2 µm are suggested for Mg implants in relation to cell adherence, cell density, and cell survival. Generally, most surface roughness in the studies were in the nm range. Some of the roughness values investigated in the reviewed studies were within the range of 1–2 µm [38,56,71,74,102], or had roughness values below 10 µm [38,71,72,74,81,93,96]. Greatly higher roughness values should be avoided, due to strong initial degradation and gas evolution.

### 5.2. Suitable Treatments for Biodegradable Mg Implants

Etching is a chemical surface treatment which is highly suitable for biodegradable Mg implants. Depending on the etching solution and the alloy used, chemical etching can vary the surface properties of the alloy. Thus, it is possible to tailor the surface roughness depending on the etching conditions. Even a minor increase in roughness (nm) showed an influence on cell adhesion [74]. Smoother surfaces were also reported to minimize the porosity of coatings. A smaller pore size in a PEO coating decreases the degradation resistance [81]. As such, etching can be used as a pre-treatment for additional coatings or as a surface modification. In addition, etched material is reported to form a stronger passivation layer compared to non-etched material, thus slowing the degradation rate [70]. Etching enables a uniform treatment over the entire surface. It can possibly be used to homogenize the surface, change the surface roughness and morphology, and remove near surface material including contamination and impurities [38,40,56]. It is suspected that it increases the degradation resistance for specific implants such as stents, rods, tubes, and screws; this is very advantageous, as it is not possible to use mechanical surface treatments such as grinding, polishing, and burnishing on these geometries. 

## 6. Conclusions

In general, this review shows that it is difficult to make reliable and clear comparisons between different studies, because several parameters and mechanisms influence the degradation behavior. One of these parameters is the amount and distribution of impurities, a factor that was not assessed in all of the investigations, although it is of critical importance. However, from this review, some rough rules can be derived:

- Considering different roughness values arising from the same type of surface treatment, especially mechanical surface treatments, a trend of increased degradation rate can be seen with higher surface roughness.
- Roughness values arising from different surface treatments are non-comparable, and thus, cannot be compared against a degradation result.
- The roughness of a Mg implant is thought to have a greater influence on initial degradation, compared to long-term degradation. The duration for implant acceptance by the body is negligibly affected by the implant’s surface roughness.
- Implant surfaces with roughness values above Sa or Ra = 0.2 µm are unsuitable for initial cell adherence and cell viability. Higher roughness should be avoided, as increased degradation is expected, and consequently, greater local alkalization will occur.
- Ca/P coatings lead to a uniform surface morphology which results in a more uniform degradation over the surface, and decreases the degradation rate compared to uncoated material. Ca/P coated Mg alloys exhibited non-toxic and biocompatible properties.
- Differences in surface roughness and additions of K_4_P_2_O_7_ or KF into the electrolyte varied the pore size of PEO coatings, which, in turn, affected the degradation rate of implant materials. A smaller pore size of the PEO coating resulted in higher degradation.
- Acid etching provides a treatment over the entire surface, removing contamination and impurities by removing surface material. In particular, acetic acid and phosphoric acid etching improved the degradation behavior, i.e., by reducing the degradation rate. Etching allows the surface properties to be tailored in order to adjust the initial and long-term degradation.

## Figures and Tables

**Table 1:** Overview of different studies concerning grinding and polishing that consider roughness and degradation behavior. * Values were determined from the diagram with the corresponding reference.

Alloy	Sample	Experiment	Solution	Time	Grinding/ Polishing	Initial Roughness	Results	Ref
Mg	Disk	pH	MEM ^1^	2 h	Polishing: 6 µm + lubricant	Ra = 0.10 µm	pH = 8.01	[74]
		Cell viability	MEM ^1^ +FBS ^2^	24 h			* CD ^3^ = 10 cells/mm^2^	
	Foil	Mass loss	DMEM ^4^ + 10% FBS ^2^	up to 80 d	1200 grit	-	Max DR ^5^ = 0.09 mg cm^−2^ d^−1^	[61]
			DI ^6^ Water				Max DR ^5^ = 0.28 mg/cmWütr²/d	
		Cell adhesion	DMEM ^4^ + 10% FBS ^2^	24 h	1200 grit	-	14.8% cell adhesion	
	Disk	Collagen quantification, cell attachment	-	2 h	180 grit	Ra = 1.89 µm	Trend of higher collagen adsorption with higher Ra, lowest CD ^3^ for highest Ra.	[6]
					800 grit	Ra = 0.29 µm		
					1200 grit	Ra = 0.15 µm		
AE44	Plate	Immersion	3.5 wt.% NaCl	4 and 12 h, 1.5 and 2.5 d	1400 grit	-	intergranular degradation started earlier after polishing	[94]
AZ31	-	PDP ^7^	0.9 wt.% NaCl	-	P1000 emery Paper	Ra = 0.33 µm	i_corr_ = 3.64 µAcm^−2^	[102]
		EIS ^8^					Rp = 934 Ωcm^2^	
	Sheet	Hydrogen	5 wt.% NaCl	24 h	1200 grit	Ra = 0.07 µm	1.11 mg/dcm²	[38]
	Disk	PDP ^7^ (1 cm^2^)	PBS ^9^	-	1200 grit	Sa = 48.58 ± 23.45 nm	i_corr_ = 34.5 ± 3.5 µA cm^−2^	[103]
							CR ^10^ = 0.76 ± 0.06 mm/y	
		Cytotoxicity	α-MEM ^11^	21 d	1200 grit	Sa = 48.58 ± 23.45 nm	* Cell survival: 92 %	
	Disk	Immersion, Hydrogen, PDP ^7^	5 wt.% NaCl	30, 200 h Immersion, 7 h Hydrogen	4000 grit	Ra = 0.2 µm	Burnishing lead to a better corrosion behavior	[39]
					Dry Burnishing			
					Cryogenic Burnishing			
	Disk	Collagen quantification, cell attachment	-	2 h	180 grit	Ra = 1.89 µm	Trend of higher collagen adsorption with higher Ra, lowest CD ^3^ for highest Ra.	[6]
					800 grit	Ra = 0.29 µm		
					1200 grit	Ra = 0.15 µm		
AZ91	-	EIS ^8^ (0.785 cm^2^)	SBF ^12^	12 h	120 grit	Sa = 0.022 µm	passivation layers on smoother surfaces last longer	[71]
					Polishing: 3 µm	Sa = 0.973 µm		
	-	PDP ^7^(0.75 cm^2^)	0.5 wt.% NaCl	-	320 grit	Sa = 0.430 µm	i_corr_ = 6.92 µA cm^−2^	[96]
					600 grit	Sa = 0.248 µm	i_corr_ = 4.79 µA cm^−2^	
					1200 grit	Sa = 0.145 µm	i_corr_ = 3.73 µA cm^−2^	
					Polishing: 3 µm	Sa= 0.08 µm	i_corr_ = 2.19 µA cm^−2^	
	Disk	PDP ^7^ (1 cm^2^)	PBS ^9^	-	1200 grit	Sa = 29.76 ± 12.69 nm	i_corr_ = 36.6 ± 3.2 µA cm^−2^	[103]
							CR ^10^ = 0.78 ± 0.07 mm/y	
		Cytotoxicity	α-MEM ^11^	21 d	1200 grit	Sa = 29.76 ± 12.69 nm	* Cell survival: 87 %	
M-4Y	Disk	Mass loss	DMEM ^4^ + 10% FBS ^2^	9.04 d	1200 grit	Ra = 65 ± 31 nm	* 89.7 %	[21]
			DI ^6^ Water				* 0.33 %	
		pH	DMEM ^4^ + 10% FBS ^2^	24 h	1200 grit	Ra = 65 ± 31 nm	* pH = 8.32	
			DI ^6^ Water				* pH = 9.00	
		Cell adhesion	DMEM ^4^ + 10% FBS ^2^		1200 grit	Ra = 65 ± 31 nm	* 22.4 %	
ZK60A	Disk	PDP (1 cm^2^)	PBS ^9^	-	1200 grit	Sa = 78.30 ± 21.63 nm	i_corr_ = 32.3 ± 2.6 µA cm^−2^	[103]
							CR ^10^ = 0.68 ± 0.01 mm/y	
		Cytotoxicity	α-MEM ^11^	21 d	1200 grit	Sa = 78.30 ± 21.63 nm	* Cell survival: 32 %	
Mg-0.5Ca-6Zn	Rectangular prism	PDP ^7^ (1 cm^2^)	Kokubo	-	2000 grit	Rq = 210 nm	i_corr_ = 365 µA cm^−2^	[104]
							CR ^10^ = 8.34 mm/y	
		Hydrogen		10 d	2000 grit	Rq = 210 nm	4.92 mL/cm²/d	
WE43	Plate		SBF ^12^	-	Polishing: 1 µm	-	i_corr_ = 642 ± 125 µA cm^−2^	[105]
Mg-1.0Ca	Rectangular prism	Mass loss	SBF ^12^	3 d	1200 grit	Sa = 4.67 nm	* 9.63 mg	[88]
		Cell viability	Extract DMEM ^4^ + 10% FBS ^2^	3 d + 4 h	1200 grit	Sa = 4.67 nm	* 100%	
		EIS ^8^ (10 × 10 mm^2^)	SBF ^12^	-	1200 grit	Sa = 4.67 nm	i_corr_ = 2.3 × 10^2^ µA cm^−2^	
Mg-0.5Sr		Mass loss	SBF ^12^	3 d	1200 grit	Sa = 2.16 nm	* 14.3 mg	
		Cell viability	Extract DMEM ^4^ + 10% FBS ^2^	3 d + 4 h	1200 grit	Sa = 2.16 nm	* 100 %	
		EIS ^8^ (10 × 10 mm^2^)	SBF ^12^	-	1200 grit	Sa = 2.16 nm	i_corr_ = 1.0 × 10^3^ µA cm^−2^	

^1^ MEM: Minimum Essential Media; ^2^ FBS: fetal bovine serum; ^3^ CD: Cell density; ^4^ DMEM: Dulbecco’s Modified Eagle’s Medium; ^5^ DR: degradation rate; ^6^ DI: deionized; ^7^ PDP: potentiodynamic polarization; ^8^ EIS: Electrochemical Impedance Spectroscopy; ^9^ PBS: phosphate buffered saline; ^10^ CR: corrosion rate; ^11^ α-MEM: MEM alpha modification Media; ^12^ SBF: simulated body fluid.

**Table 2:** Overview of different studies investigating SFF and machining and their influence on the degradation behavior. * Values were determined from the diagram with the corresponding reference.

Alloy	Sample	Experiment	Solution	Time	Machining	Initial Roughness	Results	Ref
Mg	-	PDP ^1^	HBSS ^2^ + HEPES ^3^	6 h	SFF ^4^	Ra =0.59 ± 0.04 µm	i_corr_ = 94.52 µAcm^−2^	[72]
						Ra = 2.68 ± 0.74 µm	i_corr_ ~ 189.04 µAcm^−2^	
						Ra = 9.12 ± 0.44 µm	i_corr_ ~ 567.12 µAcm^−2^	
		Mass loss				Ra =0.59 ± 0.04 µm	2.74 mg cm^−2^ d^−1^	
						Ra = 2.68 ± 0.74 µm	28.43 mg cm^−2^ d^−1^	
						Ra = 9.12 ± 0.44 µm	130.12 mg cm^−2^ d^−1^	
	Foil	Mass loss	DMEM ^5^ + 10% FBS ^6^ + P/S ^7^	80 d	Rolling	-	Max DR ^8^ = 1.2 mg cm^−2^ d^−1^	[61]
			DI ^9^ Water				Max DR ^8^ = 0.14 mg cm^−2^ d^−1^	
		Cell adhesion	DMEM ^5^ + 10% FBS ^6^ + P/S ^7^	24 h	Rolling	-	13.6 % cell adhesion	
AZ31	Sheet	Hydrogen	5 wt.% NaCl	1.5 h	Milling	Ra = 2.02 µm	54.23 mg/dcm²	[38]
				0.25 h	HT ^10^ + SB60 ^11^		563.49 mg/dcm²	
	-	PDP ^1^	0.9 wt.% NaCl	-	SP ^12^ 0.042 mmN	Ra = 1.58 µm	i_corr_ = 416.17 µAcm^−2^	[102]
					SP ^12^ 0.140 mmN	Ra = 1.72 µm	i_corr_ = 882.77 µAcm^−2^	
					SP ^12^ 0.262 mmN	Ra = 1.95 µm	i_corr_ = 1136.5 µAcm^−2^	
	Sheet	Hydrogen	5 wt.% NaCl	6.55 h	Rolling	-	* CR ^13^ = 7.17 mg cm^−2^ d^−1^	[95]
	Plate	PDP ^1^	3.5 wt.%	-	SB40 ^14^	-	i_corr_ = 2.1 µA cm^−2^	[80]
M-4Y	Disk			217 h	EDM ^15^	Ra = 196 ±47 nm	* 75.2 %	[21]
			DI ^9^ Water				* 45.9 %	
				24 h	EDM ^15^	Ra = 196 ±47 nm	* pH = 8.48	
			DI ^9^ Water				* pH = 8.98	
		Cell adhesion	DMEM ^5^ + 10% FBS ^6^ + P/S ^7^		EDM ^15^	Ra = 196 ±47 nm	* 7.82 %	
Mg-3.0Ca	Cylinder	Mass loss From hydrogen generation	0.9 wt.% NaCl	93 h	Turning: ap = 0.5 mm, vc = 10 m/min, f = 0.1 mm	* Rz = 4.48 µm	* 0.89 g/cm²	[108]
					Turning: ap = 0.5 mm, vc = 100 m/min, f = 0.1 mm	* Rz = 3.75 µm	* 1.35 g/cm²	
					Turning: ap = 0.5 mm, vc = 100 m/min, f = 0.05 mm	* Rz = 2.17 µm	* 1.29 g/cm²	
				240 h	Deep Rolling ^16^: Fr = 50 N	* Rz = 1.26 µm	* 0.07 g/cm²	
					Deep Rolling ^16^: Fr = 200 N	* Rz = 0.91 µm	* 0.02 g/cm²	
					Deep Rolling ^16^: Fr = 500 N	* Rz = 1.26 µm	* 0.02 g/cm²	
	Cylinder	Hydrogen evolution	0.9 wt.% NaCl	29 h	Turning: ap = 200 µm, vc = 100 m/min, f = 0.1 mm	* Rz = 3.98 µm	* 20.2 mL/cm²	[111]
					Deep Rolling ^16^: Fr = 50 N	* Rz = 0.63 µm	* 1.27 mL/cm²	
					Deep Rolling ^16^: Fr = 200 N	* Rz = 0.47 µm	* 0.76 mL/cm²	
		µ-CT	0.9 wt.% NaCl	29 h	Turning: ap = 200 µm, vc = 100 m/min, f = 0.1 mm	* Rz = 3.98 µm	* PV ^17^ = 19.6 mL	
					Deep Rolling ^16^: Fr = 50 N	* Rz = 0.63 µm	* PV ^17^ = 1.44 mL	
					Deep Rolling ^16^: Fr = 200 N	* Rz = 0.47 µm	* PV ^17^ = 1.05mL	
Mg-0.8Ca	Cylinder	Hydrogen evolution	0.9 wt.% NaCl	29 h	Turning: ap = 200 µm, vc = 100 m/min, f = 0.1 mm	* Rz = 4.00 µm	* 6.18 mL/cm²	[111]
					Deep Rolling ^16^: Fr = 50 N	* Rz = 0.44 µm	* 5.42 mL/cm²	
					Deep Rolling ^16^: Fr = 200 N	* Rz = 0.76 µm	* 6.22 mL/cm²	
		µ-CT	0.9 wt.% NaCl	29 h	Turning: ap = 200 µm, vc = 100 m/min, f = 0.1 mm	* Rz = 4.00 µm	* PV ^17^ = 16.3 mL	
					Deep Rolling ^16^: Fr = 50 N	* Rz = 0.44 µm	* PV ^17^ = 12.1 mL	
					Deep Rolling ^16^: Fr = 200 N	* Rz = 0.76 µm	* PV ^17^ = 6.71 mL	
		Rabbit, µ-CT	-	3 and 6 months	Turning	Ra = 3.65 µm	Turning lead to the lowest gas evolution and decomposition	[93]
					Sand milling	Ra = 32.7 µm		
					Threading	-		
Mg-5Gd	Disk	Mass loss	DMEM ^5^ + 10% FBS ^6^ + P/S ^7^	30 d	Milling	Sa =1.6 µm	CR ^13^ = 0.50 µm/d	[57]

^1^ PDP: potentiodynamic polarization; ^2^ HBBS: Hank’s Balanced Salt Solution; ^3^ HEPES: Biological buffer for cell culture media; ^4^ SFF: indirect solid free-form fabrication; ^5^ DMEM: Dulbecco’s Modified Eagle’s Medium; ^6^ FBS: fetal bovine serum; ^7^ P/S: Penicillin/Streptomycin; ^8^ DR: degradation rate; ^9^ DI: deionized; ^10^ HT: heat treated at 450 °C for 10 min (tempering); ^11^ SB60: sandblasting with glass bead type MS-6 at 60 psi; ^12^ SP: shot peening; ^13^ CR: corrosion rate; ^14^ SB40: sand blasting by #40 SiO_2_; ^15^ EDM: wire electrical discharge machining; ^16^: Deep Rolling: with vr = 25 m/min and Fr = 0.1 mm.

**Table 3:** List of different studies on acid etching being used as a surface treatment. * Values were determined from the diagram with the corresponding reference.

Alloy	Sample	Experiment	Solution	Time	Acid Etching	Initial Roughness	Results	Ref
AZ31	Sheet	SST ^1^	5 wt.% NaCl	48 h	50 g/L H_2_SO_4_^2^, 15s	* Ra = 0.98 µm	CR ^3^ = 2.20 ± 0.18 mm/y	[56]
					80 g/L HNO_3_^4^, 120 s	* Ra = 0.23 µm	CR ^3^ = 0.51 ± 0.10 mm/y	
					80 g/L H_3_PO_4_^5^, 60s	* Ra = 0.49 µm	CR ^3^ = 0.74 ± 0.31 mm/y	
		SST ^1^	5 wt.% NaCl	48 h	300 g/L CH_3_COOH ^6^, 120s	* Ra = 0.61 µm	CR ^3^ = 0.34 ± 0.08 mm/y	[40]
					80 g/L C_2_H_2_O_4_^7^, 30s	* Ra = 0.48 µm	CR ^3^ = 0.59 ± 0.11 mm/y	
					80 g/L C_6_H_8_O_7_^8^, 60s	* Ra = 0.34 µm	CR ^3^ = 0.72 ± 0.07 mm/y	
		Hydrogen	5 wt.% NaCl	~48 h	20% CH_3_COOH ^6^, 30 s	-	* CR ^3^ = 0.70 mg cm^−2^ d^−1^	[95]
				~30 h	50% H_3_PO_4_^5^, 30 s		* CR ^3^ = 1.58 mg cm^−2^ d^−1^	
				~11 h	3.3% HNO_3_^4^, 20 s		* CR ^3^ = 4.59 mg cm^−2^ d^−1^	
				~23 h	12% HF ^9^, 1200 s		* CR ^3^ = 1.68 mg cm^−2^ d^−1^	
		Hydrogen	5 wt.% NaCl	24 h	HT ^10^ + 10 % H_2_SO_4_^2^, 20 s	Ra = 2.50 µm	0.97 mg/dcm²	[38]
	Foil	Immersion	SBF ^11^	14 d	90% H_3_PO_4_^5^, 30 s	-	* CR ^3^ = 8.27 mg/d	[70]
Mg-5Gd	Disk	Mass loss	DMEM ^12^ + 10% FBS ^13^ + P/S ^14^	30 d	150 g/L CH_3_COOH ^6^, 150 s	Sa = 6.3 µm	CR ^3^ = 0.31 µm/d	[57]
					250 g/L CH_3_COOH ^6^, 150s	Sa = 5.6 µm	CR ^3^ = 0.30 µm/d	
					300 g/L CH_3_COOH ^6^, 90 s	Sa = 2.3 µm	CR ^3^ = 0.30 µm/d	

^1^ SST: salt spray test; ^2^ H_2_SO_4_: sulfuric acid; ^3^ CR: corrosion rate; ^4^ HNO_3_: nitric acid; ^5^ H_3_PO_4_: phosphoric acid; ^6^ CH_3_COOH: acetic acid; ^7^ C_2_H_2_O_4_: oxalic acid; ^8^ C_6_H_8_O_7_: citric acid; ^9^ HF: hydrofluoric acid; ^10^ HT: heat treated at 450 °C for 10 min (tempering); ^11^ SBF: simulated body fluid; ^12^ DMEM: Dulbecco’s Modified Eagle’s Medium; ^13^ FBS: fetal bovine serum; ^14^ P/S: Penicillin/Streptomycin.

**Table 4:** Overview of different Coatings studies involving roughness and degradation behavior. * Values were determined from the diagram with the corresponding reference.

Alloy	Sample	Experiment	Solution	Time	Coatings	Initial Roughness	Results	Ref
Mg	Disk	pH	MEM ^1^	2 h	Polished + NaOH	Ra = 0.23 µm	pH = 7.88	[74]
					Polished + M-SBF ^2^	Ra = 1.12 µm	pH = 8.96	
		Cell viability	MEM ^1^ + FBS ^3^	24 h	Polished + NaOH	Ra = 0.23 µm	* CD ^4^ = 177 cells/mm^2^	
					Polishing + M-SBF ^2^	Ra = 1.12 µm	* CD ^4^ = 838 cells/mm^2^	
AZ31	Foil	Immersion	SBF ^4^	2 weeks	90% H_3_PO_4_^5^, 30 s + Ca/P	-	* CR ^6^ = 7.27 mg/d	[70]
					90% H_3_PO_4_^5^, 30 s + PLA ^7^		* CR ^6^ = 6.17 mg/d	
					90% H_3_PO_4_^5^, 30 s + poly (DTH ^8^ carbonate)		* CR ^6^ = 3.83 mg/d	
	-	PDP ^9^	0.9 wt.% NaCl	-	P1000 Ground + DCPD ^10^	Ra = 4.29 µm	i_corr_ = 1.57 µA cm^−2^	[102]
					SP ^11^ 0.042 mmN + DCPD ^10^	Ra = 2.89 µm	i_corr_ = 20.03 µA cm^−2^	
					SP ^11^ 0.140 mmN + DCPD ^10^	Ra = 3.25 µm	i_corr_ = 21.96 µA cm^−2^	
					SP ^11^ 0.262 mmN + DCPD ^10^	Ra = 5.07 µm	i_corr_ = 38.12 µA cm^−2^	
	disk	PDP ^9^ (1 cm^2^)	PBS ^12^	-	1200 grit + anodizing	Sa = 49.0 ± 10.2 nm	i_corr_ = 2.72 ± 0.8 µA cm^−2^	[103]
							CR ^6^ = 0.06 ± 0.01 mm/y	
		Cytotoxicity	MEM ^1^ alpha modification Media	21 d	1200 grit + anodizing	Sa = 49.0 ± 10.2 nm	* Cell survival: 67 %	
	Plate	PDP ^9^	3.5 wt.%	-	SB ^13^ + Al ASC ^14^	Ra = 11.6 µm	i_corr_ = 2.4 × 10^2^ µA cm^−2^	[80]
					SB ^13^ + Al ASC ^14^ + PHP ^15^ (800 MPa)	Ra = 4.89 µm	-	
					SB ^13^ + Al ASC ^14^ + PHP ^15^ (1600 MPa)	-	i_corr_ = 0.8 µA cm^−2^	
					SB ^13^ + Al ASC ^14^ + PHP ^15^ (2000 MPa)	Ra = 1.12 µm	-	
					SB ^13^ + Al ASC ^14^ + PHP ^15^ + 7 wt.% oxalic acid anodizing	-	i_corr_ = 3.7 × 10^−2^ µA cm^−2^	
AZ91	Plate	PDP ^9^	3.5 wt.% NaCl	-	PEO ^16^ without K_4_P_2_O_7_	-	i_corr_ = 19.6 µA cm^−2^	[73]
					PEO ^16^ + 0.03 mol/L K_4_P_2_O_7_		i_corr_ = 1.22 × 10^−2^ µA cm^−2^	
					PEO ^16^ + 0.06 mol/L K_4_P_2_O_7_		i_corr_ = 2.27 µA cm^−2^	
					PEO ^16^ + 0.15 mol/L K_4_P_2_O_7_		i_corr_ = 4.77 µA cm^−2^	
	Plate	PDP ^9^	3.5 wt.% NaCl	-	Polishing 0.5 µm Al_2_O_3_ + PEO ^16^	Ra = 0.5 µm	i_corr_ = 7.26 × 10^−3^ µA cm^−2^	[81]
					1000 grit + PEO ^16^	Ra = 1.0 µm	i_corr_ = 5.17 × 10^−2^ µA cm^−2^	
					100 grit + PEO ^16^	Ra = 2.5 µm	i_corr_ = 0.38 µA cm^−2^	
	Plate				1000 grit + PEO ^16^ without KF ^17^	-	Rp = 8.28 × 10^3^ mΩm²	[79]
					1000 grit + PEO + KF ^17^		Rp = 4.67 × 10^3^ mΩm²	
	disk	PDP ^9^ (1 cm^2^)	PBS ^12^	-	1200 grit + anodized	Sa = 204.8 ± 62.7 nm	i_corr_ = 2.50 ± 0.5 µA cm^−2^	[103]
							CR ^6^ = 0.05 ± 0.01 mm/y	
		Cytotoxicity	MEM ^1^ alpha modification Media	21 d	1200 grit + anodized	Sa = 204.8 ± 62.7 nm	* Cell survival: 102 %	
ZK60A	disk	PDP ^9^ (1 cm^2^)	PBS ^12^	-	1200 grit + anodizing	Sa = 75.88 ± 34.49 nm	i_corr_ = 1.86 ± 0.2 µA cm^−2^	[103]
							CR ^6^ = 0.04 ± 0.01 mm/y	
		Cytotoxicity	MEM alpha modification Media	21 d	1200 grit + anodizing	Sa = 75.88 ± 34.49 nm	* Cell survival: 30 %	
Mg-0.5Ca-6Zn	Rectangular prism	PDP ^9^ (1 cm^2^)	Kokubo	-	2000 grit + 40% HF ^18^	Rq = 280 nm	i_corr_ = 6.20 µA cm^−2^	[104]
							CR ^6^ = 0.14 mm/y	
					2000 grit + DCPD ^10^/MgF_2_	Rq = 395 nm	i_corr_ = 5.72 µA cm^−2^	
							CR ^6^ = 0.13 mm/y	
					2000 grit + HA/MgF_2_	Rq = 468 nm	i_corr_ = 5.23 µA cm^−2^	
							CR ^6^ = 0.11 mm/y	
		Hydrogen	Kokubo	240 h	2000 grit + 40% HF ^18^	Rq = 280 nm	1.31 mL/cm²/d	
					2000 grit + DCPD ^10^/MgF_2_	Rq = 395 nm	1.12 mL/cm²/d	
					2000 grit + nano-(HA ^19^/MgF_2_^)^	Rq = 468 nm	0.85 mL/cm²/d	

^1^ MEM: Minimum Essential Media; ^2^ M-SBF: modified simulated body fluid; ^3^ FBS: fetal bovine serum; ^4^ CD: Cell density; ^5^ H_3_PO_4_: phosphoric acid; ^6^ CR: corrosion rate; ^7^ PLA: polymer poly(L-lactic acid); ^8^ DTH: desaminotyrosyl tyrosine hexyl; ^9^ PDP: potentiodynamic polarization; ^10^ DCPD: dicalcium phosphate dihydrate; ^11^ SP: Shot peening; ^12^ PBS: phosphate buffered saline; ^13^ SB: Sand blasting; ^14^ ASC:arc-spray coating; ^15^ PHP: post hot pressing; ^16^ PEO: plasma electrolytic oxidation; ^17^ KF: potassium fluoride; ^18^ HF: hydrofluoric acid; ^19^ HA: hydroxyapatite.

**Table 5:** Summary of different studies concerning the influence of ion implantation on the degradation behavior. * Values were determined from the diagram with the corresponding reference.

Alloy	Sample	Experiment	Solution	Time	Implantation	Initial roughness	Results	Ref
WE43	Plate	PDP ^1^	SBF ^2^	-	Polishing: 1 µm + Si ion plasma	-	i_corr_ = 27 ± 32 µA cm^−2^	[105]
Mg-1.0Ca	Rectangular prism	Mass loss	SBF ^2^	3 d	1200 grit + Zr	Sa = 5.34 nm	* 8.03 mg	[88]
					1200 grit + ZrO	Sa = 9.42 nm	* 6.77 mg	
		Cell viability	Extract assay (DMEM ^3^)	24 h + 72 h + 4 h	1200 grit + Zr	Sa = 5.34 nm	* 101 %	
					1200 grit + ZrO	Sa = 9.42 nm	* 103 %	
		EIS ^4^ (10 × 10 mm^2^)	SBF ^2^	-	1200 grit + Zr	Sa = 5.34 nm	i_corr_ = 1.2 × 10^2^ µA cm^−2^	
					1200 grit + ZrO	Sa = 9.42 nm	i_corr_ = 2.6 × 10^1^ µA cm^−2^	
Mg-0.5Sr	Rectangular prism	Mass loss	SBF ^2^	3 d	1200 grit + Zr	Sa = 4.61 nm	* 13.7 mg	[88]
					1200 grit + ZrO	Sa = 7.29 nm	* 8.52 mg	
		Cell viability	Extract assay (DMEM ^3^)	24 h + 72 h + 4 h	1200 grit + Zr	Sa = 4.61 nm	* 110 %	
					1200 grit + ZrO	Sa = 7.29 nm	* 126 %	
		EIS ^4^ (10 × 10 mm^2^)	SBF ^2^	-	1200 grit + Zr	Sa = 4.61 nm	i_corr_ = 2.5 × 10^2^ µA cm^−2^	
					1200 grit + ZrO	Sa = 7.29 nm	i_corr_ = 1.7 × 10^2^ µA cm^−2^	

^1^ PDP: potentiodynamic polarization; ^2^ SBF: simulated body fluid; ^3^ DMEM: Dulbecco’s Modified Eagle’s Medium; ^4^ EIS: Electrochemical Impedance Spectroscopy
